# Two Cases of Bacillus cereus Sepsis During Hospitalization for Hyperemesis Gravidarum

**DOI:** 10.7759/cureus.87402

**Published:** 2025-07-07

**Authors:** Megumi Shibata, Nobuko Sakai, Aya Masunaga, Shinichi Hoshi

**Affiliations:** 1 Obstetrics and Gynecology, Tokyo Riverside Hospital, Tokyo, JPN; 2 General Internal Medicine, Tokyo Riverside Hospital, Tokyo, JPN

**Keywords:** bacillus cereus, catheter-related bloodstream infection (crbsi), hospital-acquired infection, maternal sepsis, pregnancy, quick sequential organ failure assessment score, severe hyperemesis gravidarum

## Abstract

We report two cases of *Bacillus cereus* sepsis in pregnant women hospitalized for severe hyperemesis gravidarum. Both patients were previously healthy and were admitted for the management of hyperemesis gravidarum. They received intravenous amino acid-containing infusions (e.g., Bfluid^®^) as part of standard supportive care. However, they subsequently developed sepsis within the same timeframe. *B. cereus*, which is resistant to heat and alcohol-based disinfectants, was identified from blood cultures and the catheter tip culture. These cases emphasize that severe hyperemesis gravidarum can induce a transient immunocompromised state, thereby increasing susceptibility to opportunistic infections. Strict adherence to hygiene protocols and vigilant monitoring are essential to prevent nosocomial *B. cereus* infections in this vulnerable population.

## Introduction

*Bacillus cereus *(*B. cereus*)is a spore-forming bacterium that is widely distributed in the environment. It is resistant to alcohol and heat, which makes it difficult to eliminate using standard laundering methods [1-3]. Nosocomial infections caused by* B. cereus *adhering to intravenous catheter linens have been reported, particularly in association with amino acid-based infusions, resulting in catheter-related bloodstream infections (CRBSIs), which are bloodstream infections in which the catheter is considered the primary source of bacteremia [4]. In such cases, the bacteria may enter the bloodstream through a peripheral venous route, leading to sepsis, especially in immunocompromised individuals, such as cancer patients or those undergoing immunosuppressive therapy [5,6].

Sepsis during hospitalization for hyperemesis gravidarum is extremely rare. There are very few reports; one case of *B. cereus *sepsis originating from a peripheral intravenous catheter has been documented in this context, and it was difficult to treat [7]. Here, we report two simultaneous cases of *B. cereus*-induced sepsis in otherwise healthy pregnant women who were hospitalized for severe hyperemesis gravidarum requiring prolonged intravenous management. These cases suggest that, in addition to pregnancy-associated immunological tolerance, malnutrition due to hyperemesis gravidarum may further compromise immune defenses, rendering otherwise healthy pregnant women vulnerable to opportunistic infections. Increased clinical awareness of this possibility may facilitate early detection and improve patient outcomes.

## Case presentation

Case 1

A 28-year-old primiparous woman was admitted at seven weeks and three days of gestation due to severe hyperemesis gravidarum, with a urine ketone level of 3+. Although the frequency of vomiting was only a few times per day, she was unable to tolerate any food intake and vomited even small amounts of fluids. Her serum albumin level was slightly decreased at 3.6 g/dL (reference range: 3.8-5.3 g/dL). She had no significant medical or family history, including any immunological disorders. She was managed with intravenous fluids, including Bfluid® Injection (Otsuka Pharmaceutical, Inc., Tokyo, Japan), an amino acid-containing solution. Despite treatment, her condition led to a weight loss of 3.3 kg over one month. Oral intake was impossible for two weeks, followed by another two weeks during which she could consume only about one-fourth of her usual meals, necessitating continued infusion therapy. Due to frequent intravenous infiltration, her catheter was replaced approximately every three days and maintained for a maximum of one week.

**Figure 1:**
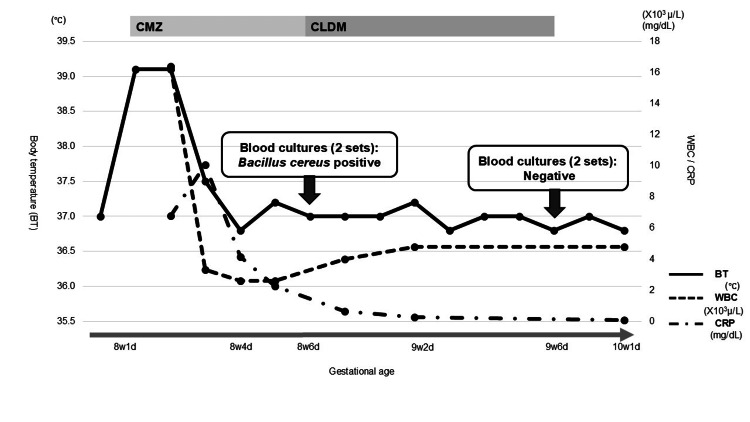
Clinical timeline of Case 1 Clinical timeline of Case 1 showing changes in WBC and CRP levels, body temperature, and major clinical interventions during early pregnancy. CMZ: cefmetazole; CLDM: clindamycin: BT: body temperature; WBC: white blood cells; NEUT: neutrophils; CRP: C-reactive protein

Figure 1 illustrates the clinical course ofCase 1, including changes in laboratory findings and treatment interventions. The specific laboratory values are summarized in Table 1 for clarity. At eight weeks and one day of gestation, she developed a fever of 39.1 °C. Her blood pressure was 102/60 mmHg, pulse rate was 90/min, and respiratory rate was 18/min. The patient presented with a headache associated with fever and nausea attributable to gravidarum hyperemesis. No clinical signs indicative of meningitis, urinary tract infection, or other apparent infectious sources were observed. Mild erythema was observed at the venous catheter insertion site; however, there were no signs of a definite infectious focus. Blood tests showed a white blood cell (WBC) count of 16,400/μL, with neutrophils accounting for 98.5%, and a C-reactive protein (CRP) level of 6.77 mg/dL. Two sets of blood cultures were collected, and her intravenous line was immediately replaced. Her quick sequential organ failure assessment (qSOFA) score was 1. No obvious infectious focus was identified at this stage. Prolonged malnutrition can lead to the translocation of commensal enteric bacteria across the intestinal mucosal barrier. In this case, the patient was initially diagnosed with bacterial translocation. Enteric Gram-negative bacilli and anaerobic bacteria are commonly implicated as primary causative organisms; therefore, empirical antibiotic therapy with cefmetazole (CMZ) was initiated. Her fever resolved rapidly following treatment. By eight weeks and four days, her WBC count had decreased to 2,600/μL (61.2% neutrophils), and CRP had declined to 2.26 mg/dL. *B. cereus *was subsequently isolated from two sets of blood cultures at eight weeks and six days of gestation. Because the patient had no abdominal symptoms and her fever disappeared after catheter exchange, a diagnosis of non-intestinal nosocomial *B. cereus *sepsis caused by the contamination of the intravenous line was made. In response, her infusion regimen was switched to a solution without amino acids, and her antibiotic was changed to clindamycin (CLDM). She remained stable and was discharged at nine weeks and six days of gestation after blood cultures turned negative.

**Table 1:** Laboratory findings in Case 1 WBC: white blood cells; NEUT: neutrophils; CRP: C-reactive protein

Laboratory Test	7w3d Admission(Baseline)	8w1d Onset of Fever	8w4d After Treatment Initiation	9w6d Blood Culture Negativity	Reference Range
WBC	6.1 (10^3^/μL)	16.4 (10^3^/μL)	2.6 (10^3^/μL)	5.0 (10^3^/μL)	3.5-9.7 (10^3^/μL)
NEUT	65.0%	98.5%	61.2%	65.3%	40%–70%
CRP	0.12 (mg/dL)	6.77 (mg/dL)	2.26 (mg/dL)	0.8 (mg/dL)	<0.3 (mg/dL)

Subsequent prenatal check-ups were unremarkable. However, as her estimated due date approached, she developed gestational hypertension. Ultimately, this led to a diagnosis of hypertensive disorders of pregnancy and the need for labor induction. At 40 weeks of gestation, she delivered a healthy female infant via vacuum-assisted vaginal delivery without maternal or neonatal complications. The infant's birth weight was 2,902 g, umbilical artery blood (UmA) pH was 7.301, and Apgar scores at one minute and five minutes were 9 and 10, respectively.

In summary, the patient was admitted for severe hyperemesis gravidarum and developed a fever on hospital day four. She was initially diagnosed with bacterial translocation and started on CMZ. However, blood cultures were positive for *B. cereus* on day five, prompting a switch to CLDM and catheter replacement. Her condition improved steadily, and she was discharged in stable condition on hospital day 12. She subsequently delivered at term without maternal or neonatal complications.

Case 2

A 30-year-old primiparous woman was admitted at six weeks and two days of gestation due to severe hyperemesis gravidarum, characterized by more than 10 episodes of vomiting per day, decreased urine output, and a urine ketone level of 3+. She had no history of immunological disorders but was undergoing treatment for depression with aripiprazole, a serotonin-norepinephrine reuptake inhibitor (SNRI), and a selective serotonin reuptake inhibitor (SSRI). She was managed with intravenous fluids, including Bfluid® as well as antiemetic medications. Her intravenous catheter required replacement every two to four days due to frequent occlusions. Despite treatment, she experienced persistent hyperemesis, resulting in a weight loss of 4 kg over one month. Due to ongoing difficulty with oral intake, she required prolonged intravenous infusion therapy.

**Figure 2:**
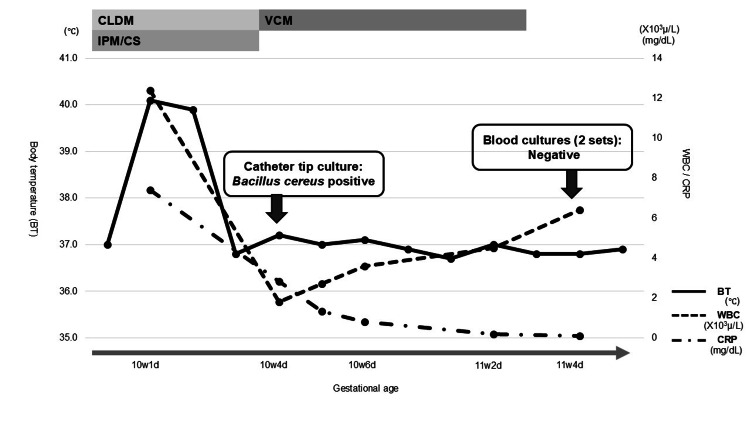
Clinical timeline of Case 2 Clinical timeline of Case 2 showing changes in WBC and CRP levels, body temperature, and major clinical interventions during early pregnancy. CLDM: clindamycin; IPM/CS: imipenem/cilastatin; VCM: vancomycin; BT: body temperature; WBC: white blood cells; NEUT: neutrophils; CRP: C-reactive protein

Figure 2 illustrates the clinical course of Case 2, including changes in laboratory findings and treatment interventions. The specific laboratory values are summarized in Table 2 for clarity. At 10 weeks and one day of gestation, she developed a fever of 40.1 °C. Her blood pressure was 82/62 mmHg, and her pulse rate was 84/min. Similar to Case 1, she exhibited no apparent clinical symptoms other than fever, and physical examination did not reveal any obvious source of infection. Notably, in this case, there was no erythema at the intravenous catheter insertion site. Blood tests showed a WBC count of 12,400/μL (neutrophils of 94.7%) and a CRP level of 7.41 mg/dL. Her qSOFA score was 1. Two sets of blood cultures and her intravenous catheter tip were submitted for microbiological analysis. Empiric antibiotic therapy with CLDM and imipenem/cilastatin (IPM/CS) was initiated, and her infusion regimen was switched to a solution without amino acids. At 10 weeks and four days, *B. cereus* was isolated from the catheter tip culture, suggesting that contamination was unlikely. Blood tests revealed a WBC count of 1,800/μL (neutrophils of 57.5%) and a CRP level of 2.83 mg/dL. As the leukopenia developed shortly after initiating IPM/CS and no other apparent causes, such as viral infection or bone marrow suppression, were identified, drug-induced leukopenia was suspected. Accordingly, the antibiotic regimen was switched to vancomycin (VCM) based on the results of susceptibility testing and its minimum inhibitory concentration (MIC), and treatment was continued until blood cultures returned negative. At 11 weeks and four days of gestation, VCM was discontinued. As her oral intake improved, she was discharged at 12 weeks and one day of gestation.

**Table 2:** Laboratory findings in Case 2 WBC: white blood cells; NEUT: neutrophils; CRP: C-reactive protein

Laboratory Test	6w2d Admission(Baseline)	10w1d Onset of Fever	10w4d After Treatment Initiation	11w4d Blood Culture Negativity	Reference Range
WBC (/μL)	6.3 (10^3^/μL)	12.4 (10^3^/μL)	1.8 (10^3^/μL)	4.7 (10^3^/μL)	3.5-9.7 (10^3^/μL)
NEUT (%)	63.0%	94.7%	57.5%	62.1%	40%–70%
CRP (mg/dL)	0.15 (mg/dL)	7.41 (mg/dL)	2.83 (mg/dL)	1.1 (mg/dL)	<0.3 (mg/dL)

At approximately 23 weeks of gestation, her fetus was diagnosed as small for gestational age (SGA), measuring -1.2 standard deviations (SD) below the mean. The fetus exhibited dolichocephaly, with a biparietal diameter (BPD) of -3.2 SD and a head circumference (HC) of -1.9 SD in the breech position, leading to underestimation of the estimated fetal weight (EFW). No structural anomalies, including intracranial abnormalities, were noted. By 28 weeks of gestation, the EFW had further declined to -2.5 SD, with a BPD of -3.7 SD and an HC of -2.3 SD. However, the fetal growth trajectory remained steady at approximately -2.5 SD. She delivered a male infant with SGA and low birth weight at 37 weeks of gestation via spontaneous breech vaginal delivery, which was complicated by placental abruption. The infant's birth weight was 2,024 g, UmA pH was 7.396, and Apgar scores at one minute and five minutes were 8 and 8, respectively.

In summary, the patient was admitted for severe hyperemesis gravidarum and developed a fever approximately one month after hospitalization. Empirical antibiotic therapy with CLDM and IPM/CS, both active against *B. cereus*, was initiated. *B. cereus* was subsequently isolated from the catheter tip on day three. Although the antibiotic regimen was later changed to VCM, her symptoms resolved, and she was discharged without complications on hospital day 14. During prenatal check-up, fetal growth restriction was observed, with EFW persistently around -2.5 SD. At 37 weeks of gestation, she delivered a male infant with SGA and LBW (2,024 g) via spontaneous breech vaginal delivery, which was complicated by placental abruption. The neonate had a favorable outcome, with Apgar scores of 8 and 8 at one minute and five minutes, and an UmA pH of 7.396.

## Discussion

We present two temporally clustered cases of *B. cereus* sepsis in pregnant women who were hospitalized in the same room for severe hyperemesis gravidarum during the summer season. Severe infections during pregnancy can rapidly compromise both maternal and fetal health, necessitating timely recognition and intervention [7,8]. Even in pregnant women without underlying comorbidities, it is essential to acknowledge their potential susceptibility to opportunistic infections. Based on the fever patterns and laboratory findings, the present cases are more appropriately diagnosed as sepsis rather than simple bacteremia. Early identification and appropriate treatment of sepsis are critical for achieving favorable perinatal outcomes.

*B. cereus* species are spore-forming bacteria widely distributed in the environment. They are resistant to alcohol and heat, making them difficult to eliminate through standard laundering methods [1-3]. Their optimal growth conditions include a temperature of approximately 30 °C and a pH range between 5.0 and 9.0, generally considered harmless environmental bacteria with low pathogenicity in healthy individuals. However, *B. cereus* species can cause CRBSI in immunosuppressed individuals [2]. A definitive CRBSI diagnosis typically requires the isolation of *B. cereus* from multiple blood cultures and from catheter tip cultures. In Case 1, two positive blood cultures, alongside the absence of other infectious foci and the clinical course, strongly suggested true *B. cereus* bacteremia rather than contamination. The antibiotic regimen was immediately changed to one effective for *B. cereus* as the cause of sepsis, discontinued the use of amino acid infusions, and replaced the intravenous catheter to physically eliminate the source of *B. cereus*. We believe that early recognition of *B. cereus* infection and prompt elimination of its favorable growth conditions contributed to successful infection control without recurrence. This was further supported by findings inCase 2, where *B. cereus* was isolated from the catheter tip culture, reinforcing the likelihood that Case 1 also represented a true infection.

Nosocomial outbreaks of *B. cereus* have been attributed to contaminated hospital linens and towels [9,10]. Moist reusable towels may serve as a reservoir for bacterial spores, increasing the risk of transmission in healthcare settings. In addition to environmental sources, glucose-amino acid infusions, such as Bfluid®, create a pH-favorable environment that facilitates bacterial proliferation, particularly during the summer months [5,6]. These solutions may inadvertently support the rapid proliferation of *B. cereus* if contamination occurs. Moreover, as a spore-forming bacterium, *B. cereus* exhibits resistance to alcohol-based disinfectants and can adhere to infusion lines [4]. This poses a significant risk when medications are administered through side ports, potentially allowing the bacteria to enter the bloodstream. While *B. cereus* is generally harmless in healthy individuals, numerous reports indicate that it can cause severe infections in patients with cancer, those undergoing chemotherapy, postoperative patients, and immunocompromised individuals [11-13].

Previous reports of sepsis during hyperemesis management have primarily implicated bacterial translocation or central venous nutrition as causative factors [5,14]. However, sepsis caused by *B. cereus* during pregnancy is extremely rare, with only one previously reported case [15]. In that case, *B. cereus* was not initially suspected as the source of fever, and the patient was treated based on other presumed causes. As a result, the patient experienced four recurrent episodes of fever by the 18th week of gestation. Pregnancy induces immunological adaptations, including suppressed NK and T cell activity and enhanced immune tolerance, rendering expectant mothers more susceptible to infections [16]. It has been suggested that dehydration and metabolic stress in hyperemesis gravidarum may impair immune cell function via vascular endothelial mechanisms, increasing susceptibility to infection and inflammation [17]. This malnourished state may exacerbate immunosuppression beyond the baseline immunotolerance of pregnancy. In Case 2, nearly one month of hospitalization for prolonged hyperemesis supports the possibility that severe or persistent hyperemesis can heighten vulnerability to opportunistic infections. Consequently, pregnant women with severe hyperemesis should be managed with the same level of infection vigilance as immunocompromised patients. Even in otherwise healthy individuals, *B. cereus *should not be readily dismissed as a contaminant when the patient is physiologically weakened. Clinical awareness is essential, as such patients may have a comparable risk profile.

To reduce the incidence of *B. cereus* sepsis in this population, a multifaceted approach to infection control is recommended.

Use of disposable towels: Moist reusable towels are particularly susceptible to *B. cereus* contamination. Replace reusable moist towels with disposable alternatives to minimize contamination risk.

Rigorous hand hygiene and skin preparation: Alcohol-based disinfectants alone may be insufficient for bacterial eradication. Hand hygiene should be thoroughly performed with soap and running water before handling intravenous lines.

Maintenance of infection line care: Bacterial proliferation has been observed at the connection sites of infusion routes. Careful disinfection and physical removal of contaminants are essential.

Environmental disinfection: Routine use of sodium hypochlorite should be incorporated into hospital cleaning protocols to ensure thorough decontamination.

Infusion management: Limit the administration of amino acid-containing infusions, such as Bfluid®, to within six hours, especially during warmer seasons [18].

Routine surveillance: As *B. cereus* can be present on healthy skin and in stool, environmental sources cannot be excluded [19]. However, include* B. cereus* in hospital microbial surveillance programs to enable early detection and timely intervention.

This report has several limitations. First, environmental cultures were not performed, making it difficult to assess the potential for environmental contamination. Second, in Case 1, the catheter tip was not submitted for culture, which limits the diagnostic certainty of catheter-related bloodstream infection. Third, detailed data such as quantitative blood cultures, time to positivity, and actual colony growth rates were unavailable, as these tests were outsourced and not accessible during the study period. These factors may limit the reproducibility and generalizability of the findings.

## Conclusions

The detection of a low-virulence organism such as *B. cereus* at the onset of sepsis suggests that the host may be in an immunocompromised state. Therefore, depending on the clinical condition of the pregnant patient, the presence of *B. cereus* should not be readily dismissed as mere contamination. The detection of a low-virulence organism indicates that more intensive treatment may be required than would ordinarily be expected. The immunological adaptations of pregnancy, coupled with the metabolic stress and nutritional deficits associated with prolonged hyperemesis, may warrant consideration of a transient immunocompromised state, increasing susceptibility to severe opportunistic infections. Consequently, the use of amino acid preparations for the treatment of hyperemesis gravidarum may increase the risk of developing life-threatening nosocomial infections, even in otherwise healthy pregnant women, particularly during the summer months when *B. cereus* proliferation is more likely.

To mitigate this risk, clinicians must maintain a high index of suspicion and recognize that patients with hyperemesis gravidarum requiring intravenous management may be predisposed to bloodstream infections. Prompt blood culture collection, thorough investigation of the infection source, and rigorous infection control measures - including strict catheter care and environmental hygiene - are essential for safeguarding both maternal and fetal health and may help prevent such avoidable infections.
